# International Homœopathic Congress

**Published:** 1896-03

**Authors:** 


### INTERNATIONAL HOMŒOPATHIC CONGRESS, 1896.

Honorary President, Dr. Dudgeon; President, Dr. Pope; Vice-President, Dr. Dyce Brown; Treasurer, Dr. J. G. Blackley; General (Permanent) Secretary, Dr. Hughes, 36 Sillwood Road, Brighton; Local Secretaries, Dr. Hawkes, 22 Abercrombie Square, Liverpool, and Mr. Dudley Wright, 55 Queen Anne Street, London, W.

In accordance with the resolutions passed at the British Homœopathic Congresses of 1894 and 1895, the following will be the arrangements for the above-mentioned gathering:

1. The Congress will be held at Queen's Hall, Langham Place, London, during the second week in July, 1896—Monday the 13th to Saturday the 18th inclusive.

2. The Congress is open to all qualified to practice medicine in their own country; and members will be at liberty to introduce visitors to the meetings at their discretion.

3. The general meetings will be held on the Tuesday, Wednesday, Thursday, and Friday, from 2.30 to 5.30 P. M., and on the Saturday at 2 P. M. Sectional meetings can be held in the Board-room of the London Homœopathic Hospital, Great Ormond Street (which has kindly been placed at the Congress' disposal for the purpose) during the forenoons, as may be arranged among the members themselves.

4. No papers will be read at the general meetings. The accepted essays will be printed and supplied to all who desire to take part in the debates on their subject-matter. They will be presented at the meetings, singly or in groups, according to their contents—a brief analysis of each being given from the chair; and the points on which they treat will then be thrown open for discussion, after an appointed opener (or openers) shall have been heard.

It will be seen from the above that our object is to discuss subjects rather than individual papers. Of the latter, therefore, we have no further need; but we should be very glad of additional communications on the topics already specified, and on those which will be later announced as chosen by the American Committee which is co-operating with us. All communications relating to the work of the Congress should be addressed to the General Secretary. The Local Secretaries will be glad to afford information relative to accommodation, etc. In connection with this it may be mentioned that the members of the British Homœopathic Society, resident in London, are being invited to open their houses, where practicable, to guests from abroad.

The President will hold a reception on the Monday evening, at the Queen's Hall, for the members of the Congress, with the ladies of their families.

---

